# Emergency Department Preparedness to Care for Sexual Assault Survivors: A Nationwide Study

**DOI:** 10.5811/westjem.59257

**Published:** 2023-04-26

**Authors:** Kristen Chalmers, Meredith Hollender, Liam Spurr, Ramya Parameswaran, Nicole Dussault, Jeanne Farnan, Sonia Oyola, Keme Carter

**Affiliations:** * University of Chicago Pritzker School of Medicine, Department of Medicine, Chicago, Illinois; † University of California San Francisco Health, San Francisco, California; ‡ Duke University, Duke University Medical Center, Department of Internal Medicine, Durham, North Carolina; § University of Chicago Pritzker School of Medicine, Department of Family Medicine, Chicago, Illinois

## Abstract

**Introduction:**

Emergency departments (ED) provide trauma-informed care to sexual assault (SA) survivors and connect them with comprehensive services. Through surveying SA survivor advocates, we aimed to 1) document updated trends in the quality of care and resources offered to SA survivors and 2) identify potential disparities according to geographic regions in the US, urban vs rural clinic locations, and the availability of sexual assault nurse examiners (SANE).

**Methods:**

We conducted a cross-sectional study between June–August 2021, surveying SA advocates who were dispatched from rape crisis centers to support survivors during ED care. Survey questions addressed two major themes in quality of care: staff preparedness to provide trauma-response care; and available resources. Staff preparedness to provide trauma-informed care was assessed through observations of staff behaviors. We used Wilcoxon rank-sum and Kruskal-Wallis tests to analyze differences in responses according to geographic regions and SANE presence.

**Results:**

A total of 315 advocates from 99 crisis centers completed the survey. The survey had a participation rate of 88.7% and a completion rate of 87.9%. Advocates who indicated that a higher proportion of their cases were attended by SANEs were more likely to report higher rates of trauma-informed staff behaviors. For example, the recalled rate of staff asking patients for consent at every step of the exam was significantly associated with SANE presence (P < 0.001). With respect to access to resources, 66.7% of advocates reported that hospitals often or always have evidence collection kits available; 30.6% reported that resources such as transportation and housing are often or always available, and 55.3% reported that SANEs are often or always part of the care team. The SANEs were reported to be more frequently available in the Southwest than in other US regions (P < 0.001) and in urban as opposed to rural areas (P < 0.001).

**Conclusion:**

Our study indicates that support from sexual assault nurse examiners is highly associated with trauma-informed staff behaviors and comprehensive resources. Urban-rural and regional disparities exist regarding access to SANEs, suggesting that elevating nationwide quality and equity in care of survivors of sexual assault requires increased investments in SANE training and coverage.

## INTRODUCTION

Sexual assault (SA) is a nationwide public health crisis with long-term health consequences. Within the United States, 43.6% of women and 24.8% of men experience some form of contact sexual violence within their lifetime.^1^ Survivors of SA bear the burden of both acute and long-lasting sequelae, including injuries, sexually transmitted diseases, and an increased risk of chronic physical and mental health problems.^2^

Emergency departments (ED) play a critical role in serving the approximately 21% of SA survivors who seek acute medical attention.^3^ Ideally, EDs provide survivors with comprehensive services to address their physical and mental health needs, including crisis counseling, sexually transmitted infection management, emergency contraception, and HIV exposure management.^4^,^5^ Survivors are also offered the option to complete a SA examination kit to obtain forensic evidence. This includes swabbing the vagina, rectum, and mouth, plucking and combing head and pubic hairs, and obtaining fingernail scrapings and blood samples.^6^,^7^ The interpersonal dynamic between ED staff and patient is critical during these invasive procedures. While positive interactions can be empowering to SA survivors, negative interactions with ED staff can increase SA survivors’ risk of post-traumatic stress symptomatology and decrease their likelihood of seeking further medical and legal assistance.^8^–^10^

Many EDs use additional support from specialized sexual assault response teams when caring for SA survivors. These teams typically consist of SA patient advocates and/or sexual assault nurse examiners (SANE). The SA patient advocates serve as first-response crisis counselors, assist survivors in navigating the medical and legal processes in the ED, and provide referrals to follow-up support services.^11^ The SA patient advocates are volunteers or staff members at sexual violence crisis centers who are dispatched to hospitals to assist with SA patient cases.^12^ SANEs are registered nurses trained in trauma-informed approaches to survivors’ medical care, conducting forensic examinations and providing forensic documentation in legal cases.^13^–^15^ Budgetary constraints, scheduling, or a lack of contracts between hospitals, SANE programs, and/or rape crisis centers lead to many SA patients receiving specialized support from only a SA advocate, a SANE, or neither.^16^

Providing SA survivors with high-quality care can be challenging for many hospitals. A 2013 survey of US hospitals found that only ~20% provided survivors with comprehensive services, including SA crisis counseling, sexually transmitted infection management, HIV management, and emergency contraception.^17^ Furthermore, small, qualitative studies suggest that ED staff have low self-efficacy when working with SA patients.^18^ Despite SA-related ED visits in the US increasing from 3,607 in 2006 to 55,296 in 2019, there is limited research documenting how EDs have responded to this increase in utilization volume.^19^ Additionally, there are no nationwide perspectives on the quality of care offered to SA patients in EDs in the wake of societal shifts such as the #MeToo movement, which has led to changes in societal perceptions of survivors and their treatment in other medical settings.^20^–^22^

**Population Health Research Capsule**

What do we already know about this issue?

*Unites States’ emergency departments provide post-assault care to over 55,000 sexual assault (SA) survivors per year. The quality of trauma-informed care and resources offered are highly variable*.

What was the research question?

*Are there disparities in the care offered to SA survivors according to geography (region and urban vs rural)?*

What was the major finding of the study?

*The availability of SA nurse examiners (SANE), which is lower in rural compared to urban areas (P<0.001), is positively associated with trauma-informed care (P<0.001) to the benefit of SA patients*.

How does this improve population health?

*Elevating nationwide quality and equity in SA survivor care requires increased investments in SANE training and coverage*.

When studying nationwide trends and potential disparities in ED care of SA survivors, patient advocates can serve as reliable sources of information.^8^ As observers of numerous SA ED cases, patient advocates have valuable insight into SA patients’ ED experiences, and their nationwide presence allows for widespread data collection. The most recent surveys of ED care of SA survivors in the US are not nationwide. For example, testimony by the US Government Accountability Office on the availability of forensic examiners was limited to data collection from six states. Therefore, more comprehensive data collection is needed. Our aim in this study, therefore, was to survey advocates to 1) document updated trends in the quality of care and resources offered to SA survivors, and 2) identify potential disparities with regard to SANE and resource availability in EDs according to US geographic regions and urban vs rural clinic locations.

## METHODS

Our methods are reported according to the Checklist for Reporting Results of Internet E-Surveys (CHERRIES).^23^

We developed an electronic survey to explore two major themes in ED preparedness in caring for SA patients: staff preparedness and physical resources. Staff preparedness to provide trauma-informed care was assessed through advocates’ observations of staff behaviors that previous studies have identified as potentially retraumatizing, such as expressing disbelief or blame and not providing thorough explanations of care.^9^,^24^,^25^ Assessment of physical resources included questions regarding how frequently hospitals had evidence collection kits available, in addition to access to resources such as transportation and emergency housing. To assess the validity of the online survey, we conducted cognitive interviews with three SA patient advocates via Zoom video call (Zoom Video Communications, San Jose, CA), and iterative changes were made to ensure survey clarity.^26^,^27^ A link to the full survey is available in Appendix 1.

Patient advocates for SA victims were recruited from participating rape crisis centers via email. We identified participating rape crisis centers via online search and contacted them via phone and email. Of 137 centers with advocacy services where study team members spoke directly to center staff, 135 agreed to distribute the survey to their SA patient advocates. After agreeing to assist with survey distribution, staff at participating centers sent the survey link and background information to their SA patient advocates via email. Before providing consent via an online survey form, participating SA patient advocates were provided information about the research aims, study time commitment, privacy risks, and investigator contact information. Participants were offered the opportunity to enter a raffle for a $250 gift card as a survey incentive.

Survey responses were captured automatically via the secure REDcap platform hosted at University of Chicago between June–August 2021. Survey data were stored separately from identifiable participant data that was collected for recruitment purposes. The survey included between 57–100 items (dependent on adaptive questioning) distributed over four pages. Survey respondents were able to review and change their answers using a back button. Surveys that were terminated early were included in the analysis.

To analyze differences in survey responses between geographic regions in the US and between urban and rural clinic locations, we first coded Likert-type survey responses on a five-point ordinal scale. Data from the US Department of Agriculture were used to classify the county within which rape crisis centers were located as urban or rural.^28^ We used non-parametric tests to assess differences in these ordinal values across comparison groups. A Wilcoxon rank-sum test was used for comparisons between two groups, and we used a Kruskal-Wallis test for comparisons of more than two groups. Correlations between two ordinal variables were assessed using a Spearman correlation. All statistical tests were two-sided and performed using R v4.0.5 (R Foundation for Statistical Computing, Vienna, Austria). Adjusted *P*-values to control the false discovery rate were computed using the Benjamini-Hochberg method; an adjusted *P*-value of < 0.05 was considered significant.^29^ The institutional review board at the study institution approved the study procedures.

## RESULTS

The survey had a participation rate (unique webpage viewers who agreed to participate out of total unique first survey-page views) of 88.7% and a completion rate (unique webpage viewers who finished the survey out of total unique views who agreed to participate) of 87.9%. A total of 321 advocates from 119 crisis centers responded to the survey. Crisis centers represented 44 states and ranged from rural crisis centers serving numerous counties to crisis centers affiliated with urban, academic medical centers. Participant demographic information is summarized in Table 1.

### Quality-of-care Trends

**Figure 1:**
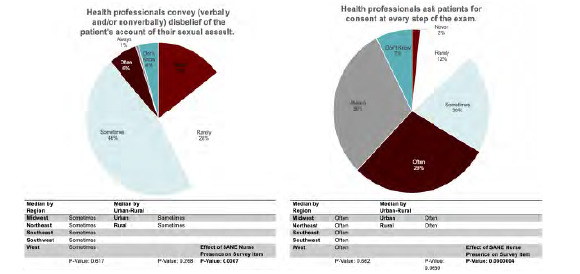
Selected quality-of-care indicators: clinician attitudes and behaviors. *SANE*, Sexual Assault Nurse Examiner.

Figure 1 presents selected quality-of-care indicators related to clinician attitudes and behaviors. Over half of respondents (53.2%) reported that they observe ED staff conveying skepticism, either verbally or non-verbally, about a patient’s account of SA sometimes, often, or always. Approximately one-quarter of respondents (28.35%) reported observing ED staff blaming survivors for the circumstances of their SA sometimes, often, or always. Similar proportions of advocates recalled that health professionals sometimes, often, or always thoroughly explain all medical care/each step of the exam and ask for consent at every step of the exam (83.6% and 78.4%, respectively), and 43.9% of advocates stated that they sometimes, often, or always recalled ED staff pressuring survivors to complete the exam or file a police report. Rates of recalled empathy were high: 95.6% of advocates reported that ED staff were sometimes, often, or always empathetic towards SA survivors.

### Quality-of-care Disparities

There were no significant differences in quality-of-care indicators related to provider attitudes and behaviors between US geographical regions or urban vs. rural regions. However, advocates who indicated that a higher proportion of their cases were attended by SANEs were more likely to report higher rates of trauma-informed staff behaviors. Notably, the recalled rate of ED staff explaining all medical care and asking patients for consent at every step of the exam was significantly associated with SANE presence (*P* < 0.001).

### Hospital Resource Trends

**Figure 2:**
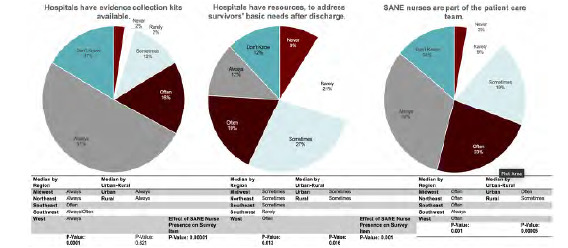
Selected hospital preparedness indicators: protocols, staff preparedness, and resources. *SANE*, Sexual Assault Nurse Examiner.

Figure 2 presents indicators of hospital preparedness, including protocols, ED staff preparedness, and resources. Indicators of procedural inefficiencies were common. A high percentage (70.7%) of advocates reported that patients sometimes, often, or always experience long wait times (>30 minutes) between different steps of their visit, including moving from the waiting room to an examination room, starting the medical forensic exam, medications, follow-up education, and discharge papers. A similar percentage of advocates (71.5%) recalled that survivors sometimes, often, or always must repeat their assault story to multiple members of the care team.

In assessing ED staff preparedness, 65.8% of advocates recalled that ED staff were sometimes, often, or always comfortable completing a medical forensic exam. A notable percentage of advocates (18.0%) reported that ED staff were never or rarely comfortable completing a medical forensic exam. While most hospitals have the resources to conduct forensic medical examinations, resources to meet survivors’ comprehensive needs are less consistent. For example, while 78.8% of advocates recalled that hospitals sometimes, often, or always have SA evidence collection kits available, only 57.9% of advocates recalled that hospitals sometimes, often, or always have resources to support patients after discharge, such as clothes for patients to change into, vouchers for follow-up care, and information to address survivors’ basic needs, such as transportation and emergency housing. Nearly three-quarters (74.4%) of advocates reported that the patient care team was sometimes, often, or always supported by a SANE.

### Hospital Resources: Geographic Disparities

With respect to differences in hospital preparedness among both US geographical regions and urban vs rural regions, SANEs were more often part of the care team in the Southwest than in other US regions (*P* < 0.001). SANEs were also more frequently present in urban as opposed to rural areas (*P* <0.001). Advocates who indicated that a higher proportion of their cases were attended by SANEs were more likely to report higher rates of several components of hospital preparedness, including shorter waiting times, lower rates of survivors repeating their story, ED staff comfort with the medical exam, availability of forensic exam kits, and availability of follow-up resources (Figure 2). SANE presence was most highly associated with ED staff being comfortable completing a medical forensic exam (*P* <0.001) and the availability of post-discharge resources (*P*< 0.001).

## DISCUSSION

Evolving societal perceptions of SA have changed the ED care of SA survivors, with increased ED utilization and advancing standards for trauma-informed care.^19^ However, our study reveals widespread variations in the quality of trauma-informed care and delivery of appropriate post-SA resources. Increased SANE presence is highly associated with more consistent observations of trauma-informed ED staff-patient interactions, as well as improved delivery of comprehensive resources to address patients’ medical and social needs. When comparing urban to rural regions of the US, patients seeking care in urban regions are more likely to be supported by a SANE.

Our findings on quality-of-care indicators related to clinician attitudes and behaviors, including levels of conveyed disbelief and blame, indicate that SA survivors who present to hospitals nationwide may be exposed to retraumatizing interactions. This study provides a nationwide perspective on the prevalence of negative interactions between SA survivors and ED staff that have been previously documented in local or regional qualitative studies.^25^,^30^,^31^ The reasons for widespread deficiencies in quality of care are multifactorial. The ED often serves as the medical safety net of communities, and patients with a myriad of acute and complex medical and social needs seek care in EDs across the country.^32^,^33^ High patient volumes, especially in the context of staffing shortages, can contribute to the deterioration in quality of care.^34^,^35^ While global improvements in patient care are a complex challenge, targeted improvement in SANE staffing can mitigate the outsized impact of negative ED encounters on survivors of SA.

The association of SANE presence with various survey measures of high-quality care aligns with prior studies documenting that EDs with SANE programs provide comprehensive medical services and proper completion of forensic examinations at higher rates than EDs lacking SANEs.^36^,^37^ Numerous studies have also demonstrated that SA survivors whose ED care is supported by SANEs are more likely to report receiving compassion, clear explanations, and choices.^38^,^39^ Our study provides an update on the trajectory of nationwide SANE coverage. In a 2009–2010 survey, approximately one-third of hospitals reported never having a SANE present during the care of SA survivors in the ED; less than 3% of advocates surveyed in our study reported never having worked with SANEs during ED management of SA survivors.^17^ This is likely the result of initiatives such as the 2018 Advanced Nursing Education - Sexual Assault Nurse Examiners Program, which allocated 24.3 million dollars of Bureau of Health Workforce of the Health Resources and Services Administration funding to SANE training at 20 academic institutions.^40^

Although SA is understudied in rural areas, our data aligns with studies documenting scarce resources, including healthcare personnel, in specific rural areas.^41^–^43^ As reported in a Pennsylvania-based study, SANEs are limited by inconsistent coverage, placing rural SA survivors at risk of receiving lower quality ED care.^44^ While our study did not find direct correlations between urban vs rural location and quality-of-care measures, SANE presence, which was less common in rural areas, was associated with many positive clinician behaviors, and their absence was associated with several negative behaviors. These negative clinician behaviors, such as conveying disbelief of the survivor’s account of sexual assault, may have serious ramifications for survivors’ legal credibility and access to resources.^45^–^47^

Addressing these urban vs rural disparities in SA survivor care requires the implementation of evidence-based strategies to recruit, train, and retain SANEs to serve rural regions. Innovative training programs developed through the Advanced Nursing Education - Sexual Assault Nurse Examiners Program have proved successful in improving SANE coverage in regions of Texas and Florida and can serve as a model for widescale SANE-coverage expansion.^48^ Blended learning programs that supplement simulated clinical experiences with online education are a promising alternative to traditional classroom learning that can be employed in rural settings.^49^

While expanding educational opportunities for SANE training is a foundational step, it is merely one component of many necessary steps to reduce disparities and elevate the quality of SA survivor care nationwide. Our study and previous work show that notable proportions of non-SANE ED staff may be uncomfortable with performing the medical forensic exam. This also provides a wider context to qualitative studies documenting low self-efficacy among ED staff when working with SA patients and demonstrates that insufficient training in SA patient care is a problem on a national level.^18^,^50^ Although there is currently no published standardized curriculum that provides continuing medical education in the trauma-informed management of SA survivors for physician trainees,^51^ educational interventions for ED staff show great promise in increasing self-efficacy and ability to avoid retraumatizing patients.^52^,^53^ Collaborative trainings that use the experience of SANEs are particularly impactful.^54^

## LIMITATIONS

The inherent limitations of this study should inform interpretation of our data. Advocates who work with centers that dispatch SA advocates to multiple hospitals reported an average of their experiences. Therefore, granularity regarding hospital type was lost. Additionally, most survey respondents were White, cisgender women. While this is likely reflective of the nationwide population of SA patient advocates, it is not reflective of SA survivors themselves and, therefore, could have influenced the survey data obtained. Future studies should further explore disparities in quality of ED care offered to SA survivors that may be influenced by patient identity. Additionally, survey respondents may have been influenced by recall bias and thus may have reported the more memorable interactions with emergency clinicians.

## CONCLUSION

Our study underscores the importance of more consistent standards for hospital preparedness to elevate the nationwide quality of ED care of sexual assault patients. Interventions should aim to decrease ED wait times, reduce the number of times patients must repeat their stories, and improve the consistency with which post-discharge resources are offered to patients. Addressing gaps in staff preparedness through more robust clinician training and increased consistency in coverage by sexual assault nurse examiners should also be prioritized to minimize potentially retraumatizing experiences for SA patients in the ED. This is particularly important to address disparities in the quality of care offered to urban and rural sexual assault survivors.

## Supplementary Information



## Figures and Tables

**Table 1:** Demographic data of survey participants.

Participant Characteristics (N = 315)	n, %
Racial/ethnic background	
White	218, 79.0%
Black	20, 7.2%
Hispanic/LatinX	41, 14.9%
Asian	10, 3.6%
Native American/Alaskan Native	5, 1.8%
Native Hawaiian /Pacific Islander	1, 0.4%
Other	4, 1.4%
Gender Identity	
Female-identifying	254, 92.4%
Male-identifying	10, 3.6%
Non-binary/gender fluid	9, 3.3%
Prefer not to say	2, 0.7%
Age (mean, standard deviation)	37.5, 13.0
Number of years of experience as advocate (mean, standard deviation)	4.6, 4.8
Number of patient experiences as a survivor advocate	
1–20	104, 37.9%
20–50	62, 22.7%
50+	108, 39.4%
