# Supplementary material for: Emergency Department Preparedness to Care for Sexual Assault Survivors: A Nationwide Study
Source: West J Emerg Med. 2023 Apr 26;24(3):629–36. doi: 10.5811/westjem.59257 (PMC10284505; doi:10.5811/westjem.59257)
Supplement: Supplementary file 1 [file wjem-24-629-Appendix-1.docx]

**Full Survey Content**

**Sexual Assault Survivor Advocate Survey**

**Research Aims:** The purpose of this research project is to formally evaluate the current practices and procedures in emergency departments for patients who have been recently sexually assaulted. The project will assess how sexual assault medical advocates perceive the treatment of sexual assault patients in the emergency department by non-SANE health professionals. The project will also seek to understand potential disparities that exist in the emergency department care of sexual assault patients. The data collected in the research project will be used to inform how we can improve emergency department protocols and provider training for sexual assault patients.

This survey should take less than 15 minutes to complete.

We will not collect any identifying information about you. Your responses are confidential, and any publications from this study responses will be reported in aggregate. Your participation is completely voluntary, and you are free to skip any question. Your response will not influence your status as a sexual assault medical advocate or any medical care or employment relationships you have with (binded). However, to ensure the validity of our findings, please try to answer all of the questions. Participating in this study will make you eligible to be entered into a raffle for a $250 Amazon gift card.

If you have questions about this, study please contact (principal investigator name and email)

Thank you in advance for your time!

**Confidentiality Clause:**

As a sexual assault advocate, you are expected to keep any and all communication with the survivors you see in the emergency room and in follow up confidential. Under no circumstances should advocates share the names of the survivors they see in the ED, or the names of the accused, with one another or on this survey. This is not only the ethical standard of conduct of sexual assault advocate volunteers and staff, but also a privileged relationship with confidential communication that is protected by Illinois law (Rape Crisis Personnel Statute 735 ILCS 5/8-802.1). Other laws may apply in your state of residence.

**Part 1 - Provider attitudes**

Part 1 asks you about statements related to health professionals' attitudes, or the impact of assumptions, biases, and emotional valence on the care they provide for survivors. Part 1 also asks if your observations have varied across your experiences as a survivor advocate according to aspects of the survivor's identity, including race/ethnicity, gender, mental health status. Throughout this survey, all references to "patients" are sexual assault patients and all references to "health professionals" are non-SANE trained health professionals. Please answer the following questions based on behaviors you have observed in the ED.

Health professionals convey disbelief of the patient's account of their sexual assault.

- Never
- Rarely
- Sometimes
- Often
- Always
- Don’t Know

Health professionals conveying (verbally and/or nonverbally) disbelief of the patient's account of their sexual assault is affected by....

|  | Does not impact conveyed disbelief from ED staff | Moderately impacts conveyed disbelief from ED staff | Greatly impacts conveyed disbelief from ED staff | I have not seen enough cases to comment on a pattern |
| --- | --- | --- | --- | --- |
| Survivor Race |  |  |  |  |
| Survivor Gender |  |  |  |  |
| Survivor Mental Health Status |  |  |  |  |
| Survivor Intoxication/Drug Use |  |  |  |  |
| Survivor Socioeconomic/Housing Status |  |  |  |  |
| Survivor Age |  |  |  |  |

*(If selected race moderately impacts provider attitudes or greatly impacts disbelief)*

Based on your observations, healthcare workers are more likely to communicate disbelief to a survivor of the following categories (please check all that apply):

- White survivors
- Black or African American survivors
- American Indian / Alaskan Native survivors
- Asian survivors
- Native Hawaiian/Pacific Islander survivors
- Hispanic survivors
- LatinX survivors
- Other

Please specify "Other"

*(If selected gender moderately impacts provider attitudes or greatly impacts disbelief)*

Based on your observations, healthcare workers are more likely to communicate disbelief to a survivor of the following categories (please check all that apply):

- Cis-female survivors
- Cis-male survivors
- Trans-female survivors
- Trans-male survivors
- Genderqueer/gender fluid survivors
- Other

Please specify "Other"

*(If selected mental health status moderately impacts provider attitudes or greatly impacts disbelief)*

Based on your observations, healthcare workers are more likely to communicate disbelief to a survivor of the following categories (please check all that apply):

- Survivors with substance use disorders
- Survivors diagnosed with psychiatric disorders (Including schizophrenia, bipolar disorder, depressive disorders, or anxiety disorders) who are not actively experiencing psychosis
- Survivors who are actively experiencing psychosis during their ED encounter
- Other

Please specify "Other"

*(If selected survivor intoxication/drug use moderately impacts provider attitudes or greatly impacts disbelief)*

Based on your observations, healthcare workers are more likely to communicate disbelief to a survivor of the following categories (please check all that apply):

- Survivors who were intoxicated (alcohol) at the time of the assault
- Survivor who intoxicated (recreational drugs) at the time of the assault
- Other

Please specify "Other"

*(If selected survivor socioeconomic/housing status moderately impacts provider attitudes or greatly impacts disbelief)*

Based on your observations, healthcare workers are more likely to communicate disbelief to a survivor of the following categories (please check all that apply):

- Survivors experiencing homelessness
- Uninsured survivors
- Survivors who are sex workers
- Other

Please specify "Other"

*(If selected survivor age moderately impacts provider attitudes or greatly impacts disbelief)*

Based on your observations, healthcare workers are more likely to communicate disbelief to a survivor of the following categories (please check all that apply):

- Survivors who are minors (< 18)
- Survivors who are elderly (65+)
- Other

Please specify "Other"

Health professionals blame the patient for the circumstances of their sexual assault.

- Never
- Rarely
- Sometimes
- Often
- Always
- Don’t Know

Health professionals blaming the patient for the circumstances of their sexual assault is affected by...

|  | Does not impact conveyed blame from ED staff | Moderately impacts conveyed blame from ED staff | Greatly impacts conveyed blame from ED staff | I have not seen enough cases to comment on a pattern |
| --- | --- | --- | --- | --- |
| Survivor Race |  |  |  |  |
| Survivor Gender |  |  |  |  |
| Survivor Mental Health Status |  |  |  |  |
| Survivor Intoxication/Drug Use |  |  |  |  |
| Survivor Socioeconomic/Housing Status |  |  |  |  |
| Survivor Age |  |  |  |  |

*(If selected race moderately impacts provider attitudes or greatly impacts blame)*

Based on your observations, healthcare workers are more likely to communicate blame to a survivor of the following categories (please check all that apply):

- White survivors
- Black or African American survivors
- American Indian / Alaskan Native survivors
- Asian survivors
- Native Hawaiian/Pacific Islander survivors
- Hispanic survivors
- LatinX survivors
- Other

Please specify "Other"

*(If selected gender moderately impacts provider attitudes or greatly impacts blame)*

Based on your observations, healthcare workers are more likely to communicate blame to a survivor of the following categories (please check all that apply):

- Cis-female survivors
- Cis-male survivors
- Trans-female survivors
- Trans-male survivors
- Genderqueer/gender fluid survivors
- Other

Please specify "Other"

*(If selected mental health status moderately impacts provider attitudes or greatly impacts blame)*

Based on your observations, healthcare workers are more likely to communicate blame to a survivor of the following categories (please check all that apply):

- Survivors with substance use disorders
- Survivors diagnosed with psychiatric disorders (Including schizophrenia, bipolar disorder, depressive disorders, or anxiety disorders) who are not actively experiencing psychosis
- Survivors who are actively experiencing psychosis during their ED encounter
- Other

Please specify "Other"

*(If selected survivor intoxication/drug use moderately impacts provider attitudes or greatly impacts blame)*

Based on your observations, healthcare workers are more likely to communicate blame to a survivor of the following categories (please check all that apply):

- Survivors who were intoxicated (alcohol) at the time of the assault
- Survivor who intoxicated (recreational drugs) at the time of the assault
- Other

Please specify "Other"

*(If selected survivor socioeconomic/housing status moderately impacts provider attitudes or greatly impacts blame)*

Based on your observations, healthcare workers are more likely to communicate blame to a survivor of the following categories (please check all that apply):

- Survivors experiencing homelessness
- Uninsured survivors
- Survivors who are sex workers
- Other

Please specify "Other"

*(If selected survivor age moderately impacts provider attitudes or greatly impacts blame)*

Based on your observations, healthcare workers are more likely to communicate blame to a survivor of the following categories (please check all that apply):

- Survivors who are minors (< 18)
- Survivors who are elderly (65+)
- Other

Please specify "Other"

Health professionals show empathy for the survivor.

- Never
- Rarely
- Sometimes
- Often
- Always
- Don’t Know

Health professionals showing empathy for the survivor is affected by...

|  | Does not impact empathy from ED staff | Moderately impacts empathy from ED staff | Greatly impacts empathy from ED staff | I have not seen enough cases to comment on a pattern |
| --- | --- | --- | --- | --- |
| Survivor Race |  |  |  |  |
| Survivor Gender |  |  |  |  |
| Survivor Mental Health Status |  |  |  |  |
| Survivor Intoxication/Drug Use |  |  |  |  |
| Survivor Socioeconomic/Housing Status |  |  |  |  |
| Survivor Age |  |  |  |  |

*(If selected race moderately impacts provider attitudes or greatly impacts empathy)*

Based on your observations, healthcare workers are more likely to display empathy

to a survivor of the following categories (please check all that apply):

- White survivors
- Black or African American survivors
- American Indian / Alaskan Native survivors
- Asian survivors
- Native Hawaiian/Pacific Islander survivors
- Hispanic survivors
- LatinX survivors
- Other

Please specify "Other"

*(If selected gender moderately impacts provider attitudes or greatly impacts empathy)*

Based on your observations, healthcare workers are more likely to display empathy to a survivor of the following categories (please check all that apply):

- Cis-female survivors
- Cis-male survivors
- Trans-female survivors
- Trans-male survivors
- Genderqueer/gender fluid survivors
- Other

Please specify "Other"

*(If selected mental health status moderately impacts provider attitudes or greatly impacts empathy)*

Based on your observations, healthcare workers are more likely to display empathy to a survivor of the following categories (please check all that apply):

- Survivors with substance use disorders
- Survivors diagnosed with psychiatric disorders (Including schizophrenia, bipolar disorder, depressive disorders, or anxiety disorders) who are not actively experiencing psychosis
- Survivors who are actively experiencing psychosis during their ED encounter
- Other

Please specify "Other"

*(If selected survivor intoxication/drug use moderately impacts provider attitudes or greatly impacts empathy)*

Based on your observations, healthcare workers are more likely to display empathy to a survivor of the following categories (please check all that apply):

- Survivors who were intoxicated (alcohol) at the time of the assault
- Survivor who intoxicated (recreational drugs) at the time of the assault
- Other

Please specify "Other"

*(If selected survivor socioeconomic/housing status moderately impacts provider attitudes or greatly impacts empathy)*

Based on your observations, healthcare workers are more likely to display empathy to a survivor of the following categories (please check all that apply):

- Survivors experiencing homelessness
- Uninsured survivors
- Survivors who are sex workers
- Other

Please specify "Other"

*(If selected survivor age moderately impacts provider attitudes or greatly impacts empathy)*

Based on your observations, healthcare workers are more likely to display empathy to a survivor of the following categories (please check all that apply):

- Survivors who are minors (< 18)
- Survivors who are elderly (65+)
- Other

Please specify "Other"

**Part 2 - Disempowering provider behaviors**

Part 2 asks you about statements related to disempowering provider behaviors, or specific provider actions that undermine patient autonomy and shared decision making. Part 2 also asks if your observations have varied across your experiences as a survivor advocate according to aspects of the survivor's identity, including race/ethnicity, gender, mental health status. All references to "patients" are sexual assault patients and all references to "health professionals" are non-SANE trained health professionals. Please answer the following questions based on behaviors you have observed in the ED.

Health professionals thoroughly explain all medical care and each step of the exam.

- Never
- Rarely
- Sometimes
- Often
- Always
- Don’t Know

Health professionals thoroughly explaining all medical care and each step of the exam is affected by...

|  | Does not impact explanations  from ED staff | Moderately impacts explanations from ED staff | Greatly impacts explanations from ED staff | I have not seen enough cases to comment on a pattern |
| --- | --- | --- | --- | --- |
| Survivor Race |  |  |  |  |
| Survivor Gender |  |  |  |  |
| Survivor Mental Health Status |  |  |  |  |
| Survivor Intoxication/Drug Use |  |  |  |  |
| Survivor Socioeconomic/Housing Status |  |  |  |  |
| Survivor Age |  |  |  |  |

*(If selected race moderately impacts provider attitudes or greatly impacts explanations)*

Based on your observations, healthcare workers are less likely to thoroughly explain all medical care and each step of the exam to survivors of the following categories (please check all that apply):

- White survivors
- Black or African American survivors
- American Indian / Alaskan Native survivors
- Asian survivors
- Native Hawaiian/Pacific Islander survivors
- Hispanic survivors
- LatinX survivors
- Other

Please specify "Other"

*(If selected gender moderately impacts provider attitudes or greatly impacts explanations)*

Based on your observations, healthcare workers are less likely to thoroughly explain all medical care and each step of the exam to survivors of the following categories (please check all that apply):

- Cis-female survivors
- Cis-male survivors
- Trans-female survivors
- Trans-male survivors
- Genderqueer/gender fluid survivors
- Other

Please specify "Other"

*(If selected mental health status moderately impacts provider attitudes or greatly impacts explanations)*

Based on your observations, healthcare workers are less likely to thoroughly explain all medical care and each step of the exam to survivors of the following categories (please check all that apply):

- Survivors with substance use disorders
- Survivors diagnosed with psychiatric disorders (Including schizophrenia, bipolar disorder, depressive disorders, or anxiety disorders) who are not actively experiencing psychosis
- Survivors who are actively experiencing psychosis during their ED encounter
- Other

Please specify "Other"

*(If selected survivor intoxication/drug use moderately impacts provider attitudes or greatly impacts explanations)*

Based on your observations, healthcare workers are less likely to thoroughly explain all medical care and each step of the exam to survivors of the following categories (please check all that apply):

- Survivors who were intoxicated (alcohol) at the time of the assault
- Survivor who intoxicated (recreational drugs) at the time of the assault
- Other

Please specify "Other"

*(If selected survivor socioeconomic/housing status moderately impacts provider attitudes or greatly impacts explanations)*

Based on your observations, healthcare workers are less likely to thoroughly explain all medical care and each step of the exam to survivors of the following categories (please check all that apply):

- Survivors experiencing homelessness
- Uninsured survivors
- Survivors who are sex workers
- Other

Please specify "Other"

*(If selected survivor age moderately impacts provider attitudes or greatly impacts explanations)*

Based on your observations, healthcare workers are less likely to thoroughly explain all medical care and each step of the exam to survivors of the following categories (please check all that apply):

- Survivors who are minors (< 18)
- Survivors who are elderly (65+)
- Other

Please specify "Other"

Health professionals ask patients for consent at every step of the exam.

- Never
- Rarely
- Sometimes
- Often
- Always
- Don’t Know

Health professionals asking for consent at every step of the exam is affected by...

|  | Does not impact ED staff asking for consent | Moderately impacts ED staff asking for consent | Greatly impacts ED staff asking for consent | I have not seen enough cases to comment on a pattern |
| --- | --- | --- | --- | --- |
| Survivor Race |  |  |  |  |
| Survivor Gender |  |  |  |  |
| Survivor Mental Health Status |  |  |  |  |
| Survivor Intoxication/Drug Use |  |  |  |  |
| Survivor Socioeconomic/Housing Status |  |  |  |  |
| Survivor Age |  |  |  |  |

*(If selected race moderately impacts provider attitudes or greatly impacts asking for consent)*

Based on your observations, healthcare workers are less likely to ask for consent at every step of the exam when the survivor belongs to the following categories (please check all that apply):

- White survivors
- Black or African American survivors
- American Indian / Alaskan Native survivors
- Asian survivors
- Native Hawaiian/Pacific Islander survivors
- Hispanic survivors
- LatinX survivors
- Other

Please specify "Other"

*(If selected gender moderately impacts provider attitudes or greatly impacts asking for consent)*

Based on your observations, healthcare workers are less likely to ask for consent at every step of the exam when the survivor belongs to the following categories (please check all that apply):

- Cis-female survivors
- Cis-male survivors
- Trans-female survivors
- Trans-male survivors
- Genderqueer/gender fluid survivors
- Other

Please specify "Other"

*(If selected mental health status moderately impacts provider attitudes or greatly impacts asking for consent)*

Based on your observations, healthcare workers are less likely to ask for consent at every step of the exam when the survivor belongs to the following categories (please check all that apply):

- Survivors with substance use disorders
- Survivors diagnosed with psychiatric disorders (Including schizophrenia, bipolar disorder, depressive disorders, or anxiety disorders) who are not actively experiencing psychosis
- Survivors who are actively experiencing psychosis during their ED encounter
- Other

Please specify "Other"

*(If selected survivor intoxication/drug use moderately impacts provider attitudes or greatly impacts asking for consent)*

Based on your observations, healthcare workers are less likely to ask for consent at every step of the exam when the survivor belongs to the following categories (please check all that apply):

- Survivors who were intoxicated (alcohol) at the time of the assault
- Survivor who intoxicated (recreational drugs) at the time of the assault
- Other

Please specify "Other"

*(If selected survivor socioeconomic/housing status moderately impacts provider attitudes or greatly impacts asking for consent)*

Based on your observations, healthcare workers are less likely to ask for consent at every step of the exam when the survivor belongs to the following categories (please check all that apply):

- Survivors experiencing homelessness
- Uninsured survivors
- Survivors who are sex workers
- Other

Please specify "Other"

*(If selected survivor age moderately impacts provider attitudes or greatly impacts asking for consent)*

Based on your observations, healthcare workers are less likely to ask for consent at every step of the exam when the survivor belongs to the following categories (please check all that apply):

- Survivors who are minors (< 18)
- Survivors who are elderly (65+)
- Other

Please specify "Other"

Health professionals pressure survivors to complete the exam or to file a police report.

- Never
- Rarely
- Sometimes
- Often
- Always
- Don’t Know

Health professionals asking for consent at every step of the exam is affected by...

|  | Does not impact pressure from ED staff | Moderately impacts pressure from ED staff | Greatly impacts pressure from ED staff | I have not seen enough cases to comment on a pattern |
| --- | --- | --- | --- | --- |
| Survivor Race |  |  |  |  |
| Survivor Gender |  |  |  |  |
| Survivor Mental Health Status |  |  |  |  |
| Survivor Intoxication/Drug Use |  |  |  |  |
| Survivor Socioeconomic/Housing Status |  |  |  |  |
| Survivor Age |  |  |  |  |

*(If selected race moderately impacts provider attitudes or greatly impacts pressure from ED staff)*

Based on your observations, healthcare workers are more likely to pressure survivors to complete the exam or file a police report when the survivor belongs to the following categories (please check all that apply):

- White survivors
- Black or African American survivors
- American Indian / Alaskan Native survivors
- Asian survivors
- Native Hawaiian/Pacific Islander survivors
- Hispanic survivors
- LatinX survivors
- Other

Please specify "Other"

*(If selected gender moderately impacts provider attitudes or greatly impacts impacts pressure from ED staff)*

Based on your observations, healthcare workers are more likely to pressure survivors to complete the exam or file a police report when the survivor belongs to the following categories (please check all that apply):

- Cis-female survivors
- Cis-male survivors
- Trans-female survivors
- Trans-male survivors
- Genderqueer/gender fluid survivors
- Other

Please specify "Other"

*(If selected mental health status moderately impacts provider attitudes or greatly impacts pressure from ED staff)*

Based on your observations, healthcare workers are more likely to pressure survivors to complete the exam or file a police report when the survivor belongs to the following categories (please check all that apply):

- Survivors with substance use disorders
- Survivors diagnosed with psychiatric disorders (Including schizophrenia, bipolar disorder, depressive disorders, or anxiety disorders) who are not actively experiencing psychosis
- Survivors who are actively experiencing psychosis during their ED encounter
- Other

Please specify "Other"

*(If selected survivor intoxication/drug use moderately impacts provider attitudes or greatly impacts pressure from ED staff)*

Based on your observations, healthcare workers are more likely to pressure survivors to complete the exam or file a police report when the survivor belongs to the following categories (please check all that apply):

- Survivors who were intoxicated (alcohol) at the time of the assault
- Survivor who intoxicated (recreational drugs) at the time of the assault
- Other

Please specify "Other"

*(If selected survivor socioeconomic/housing status moderately impacts provider attitudes or greatly impacts pressure from ED staff)*

Based on your observations, healthcare workers are more likely to pressure survivors to complete the exam or file a police report when the survivor belongs to the following categories (please check all that apply):

- Survivors experiencing homelessness
- Uninsured survivors
- Survivors who are sex workers
- Other

Please specify "Other"

*(If selected survivor age moderately impacts provider attitudes or greatly impacts pressure from ED staff)*

Based on your observations, healthcare workers are more likely to pressure survivors to complete the exam or file a police report when the survivor belongs to the following categories (please check all that apply):

- Survivors who are minors (< 18)
- Survivors who are elderly (65+)
- Other

Please specify "Other"

What is your age?

What year did you start volunteering/working as a sexual assault advocate?

Approximately how many patients have you worked with as a survivor advocate?

- 1-10
- 11-20
- 21-30
- 31-40
- 41-50
- 50+

What percentage of cases that you have attended had a SANE nurse present?

- 0-25%
- 26-50%
- 51-75%
- 75-100%

What is your gender identity?

- Male-identifying
- Female-identifying
- Non-binary / Genderfluid
- Other
- Prefer not to say

If you selected "Other" please specify:

What race/ethnicity do you identify with (Please select all that apply):

- White
- Black or African American
- American Indian / Alaskan Native
- Asian
- Native Hawaiian/Pacific Islander
- Hispanic
- LatinX
- Other

If you selected "Other" please specify:

Outside of your work with as a sexual assault survivor advocate, do you work in healthcare?

- Yes
- No

What organization do you volunteer/work with?
